# Effects of Working Memory Capacity on Metacognitive Monitoring: A Study of Group Differences Using a Listening Span Test

**DOI:** 10.3389/fpsyg.2016.00285

**Published:** 2016-03-01

**Authors:** Mie Komori

**Affiliations:** Department of Cross-cultural Studies in Japan, Osaka University of TourismOsaka, Japan

**Keywords:** working memory, metacognition, monitoring, working memory capacity, individual differences

## Abstract

Monitoring is an executive function of working memory that serves to update novel information, focusing attention on task-relevant targets, and eliminating task-irrelevant noise. The present research used a verbal working memory task to examine how working memory capacity limits affect monitoring. Participants performed a Japanese listening span test that included maintenance of target words and listening comprehension. On each trial, participants responded to the target word and then immediately estimated confidence in recall performance for that word (metacognitive judgment). The results confirmed significant differences in monitoring accuracy between high and low capacity groups in a multi-task situation. That is, confidence judgments were superior in high vs. low capacity participants in terms of absolute accuracy and discrimination. The present research further investigated how memory load and interference affect underestimation of successful recall. The results indicated that the level of memory load that reduced word recall performance and led to an underconfidence bias varied according to participants' memory capacity. In addition, irrelevant information associated with incorrect true/ false decisions (secondary task) and word recall within the current trial impaired monitoring accuracy in both participant groups. These findings suggest that interference from unsuccessful decisions only influences low, but not high, capacity participants. Therefore, monitoring accuracy, which requires high working memory capacity, improves metacognitive abilities by inhibiting task-irrelevant noise and focusing attention on detecting task-relevant targets or useful retrieval cues, which could improve actual cognitive performance.

## Introduction

Remarkable progress has been made using the concept of working memory (WM) by Baddeley and Hitch (1974) to explain how people differ in their cognitive abilities. Daneman and Carpenter (1980) created procedures for measuring working memory capacity (WMC), such as the reading span test (RST) and the listening span test (LST), and demonstrated a significant correlation between WMC and language comprehension. Furthermore, WMC is assumed to be relatively stable within a person and independent of the specific language (Osaka and Osaka, 1992) or task (Turner and Engle, 1989). Conway and colleagues reviewed the methodological merits of WM span tests as research tools, and noted that executive attentional processes are critical components of the WM span tests, including the RST (Conway et al., 2005). This may be why WMC predicts many cognitive abilities.

Because Baddeley (1986) accepted the Supervisory Attentional System proposed by Norman and Shallice (1986) as a basis for the central executive component, many studies on WMC have focused on attentional control with limited processing resources (e.g., Just and Carpenter, 1992; Conway and Engle, 1996; Cowan, 1999; Osak et al., 2002; Engle and Kane, 2004; Kane et al., 2007; for reviews see Baddeley, 2007; Chow and Conway, 2015). Baddeley (2007) conceptualized the central executive components in relation to neuropsychological evidence from patients with frontal-lobe damage, as abilities to focus, divide, and switch attention. Cowan (1999) also developed an attentional theory of WM that consisted of activated LTM and attentional focus. Although Cowan's (1999) model is structurally different from Baddeley's (2000), which contains more specific storage properties, both agree that attentional focus plays a critical role in WMC (Baddeley, 2012; Cowan et al., 2014).

Other research on WM explained attentional focus as the ability to maintain or inhibit information (e.g., Engle and Kane, 2004; Miyake and Friedman, 2012). In the framework used by Engle and colleagues, one function of executive control is to actively maintain the task goal, and the other is to resolve response competition or conflict by blocking goal-irrelevant representations or responses (Engle and Kane, 2004; Kane et al., 2007). Unsworth and Engle (2007) developed a dual-component model that contains a process for maintaining goal-relevant information in an active state (primary memory), and a process for retrieving cue-dependent information in the presence of irrelevant information (secondary memory). This model assumes that individual difference in WMC can come from both active maintenance in primary memory and controlled retrieval in secondary memory. Similar to Engle's framework, Miyake and colleagues assumed that common executive function (EF) reflects active maintenance of task goals, goal-relevant information, and response inhibition (also see Miyake et al., 2000; Friedman and Miyake, 2004; Miyake and Friedman, 2012).

Constant monitoring of WM representations is essential for adding new information related to the task goal and eliminating noise, that is, for triggering attentional control processes like focusing (Osak et al., 2002) and updating (Miyake et al., 2000). Although, most researchers assume that monitoring underlies control, monitoring has not been a central topic in WM studies.

Monitoring plays an important role in the literature on metacognition, which refers to knowledge and experience about one's own cognitive phenomena (Flavell, 1979). Nelson and Narens (1994) proposed a framework that focused on both cognition and metacognition with two levels of information processes: the object-level and meta-level. In this framework, monitoring processes evaluate ongoing task performance, which is represented as bottom-up information flow from the object-level to the meta-level. Based on the monitored information, top-down flow from control processes conveys commands for continuation, breaking, or modification from the meta-level to object-level processor.

To measure the function of monitoring in cognitive processes, metacognitive judgments are used: ease of learning, judgments of learning (JOLs), source-monitoring and retrospective confidence judgments (Nelson and Narens, 1994; Nelson, 1996; see also Dunlosky and Metcalfe, 2009). When participants failed to recall target items, feeling of knowing (FOK) and/or the tip of the tongue (TOT) were evaluated (e.g., Schwartz, 2008). Many previous studies have examined metacognitive judgments in a variety of cognitive tasks, including paired-associate learning (Nelson and Dunlosky, 1991; Nelson and Narens, 1994; Nelson et al., 2004), general knowledge (Koriat et al., 1980; Schwartz, 2008), and sentence comprehension (Thiede et al., 2003; Maki et al., 2005).

As for the bases of metacognition judgments, Koriat (2012) classified theoretical approaches as follows. The direct-access approach proposes that metacognitive judgments depend on the existence and strength of stored memory traces. The information-based approach focuses on an analytic inference weighing the pros and cons of information in memory to reach a metacognitive judgment. The experience-based approach assumes that mnemonic cues derived online from task performance directly evoke a metacognitive feeling. Put another way, monitoring is supposed to survey WM representations to detect (a) the existence and strength of target traces, (b) the outcome of probability inference, or (c) available mnemonic cues.

Considered within the dual-component model of WM (Unsworth and Engle, 2007), direct access to target memory traces can be equivalent to monitoring the contents of primary memory related to a task goal. The Miyake and Friedman's (2012) unity/diversity model also supports the active maintenance of goal-relevant information, in other words, target traces, with the common EF mechanism. In object-level cognitive processes, direct access to target traces is essential to accomplish a task goal.

In meta-level monitoring, detecting mnemonic cues through peripheral information is more important than the target trace *per se* (Schwartz, 1994; Koriat, 2012). For instance, JOLs and retrospective confidence judgments decrease with learning time and retrieval time, respectively (Koriat et al., 2006). Such inference processes in the information- or experience-based approach require controlled retrieval from secondary memory, as suggested by Unsworth and Engle (2007). This metacognitive process corresponds to the updating and monitoring function proposed by Miyake and Friedman (2012) that can manage information gating and retrieval from non-target secondary memory. Therefore, the accuracy of metacognitive judgments is assumed to depend on the ability to find effective cues and ignore goal-irrelevant information.

The previous functional magnetic resonance imaging (fMRI) studies on WM discovered that the prefrontal cortex (PFC) plays a crucial role in EF (for reviews see Shimamura, 2008; D'Esposito and Postle, 2015). In addition, the basis of individual differences in verbal WMC was reported to be associated with the neural network between the dorsolateral prefrontal cortex (DLPFC) and the anterior cingulate cortex (ACC), in coordination with the superior parietal lobule (SPL) activity especially for focusing (Osaka et al., 2003, 2004, 2007). According to Shimamura (2008), which reviewed findings from neurocognitive studies of WM and metacognition, various kinds of metacognitive judgments should also depend on PFC activity, and PFC should include metacognitive monitors and controllers for object-level cognition. The ventrolateral PFC is involved in stimulus selection, the ACC is involved in conflict monitoring, and the DLPFC is involved in updating and rerouting (Shimamura, 2008).

Schwartz (2008) provided experimental data suggesting that metacognition shares processes and resources with object-level cognition. Schwartz examined FOK and TOT processes under WMC constraints using a dual-task method in which participants had to maintain verbal information (four or six digits) or visual information (a shape in a particular color) while answering general knowledge questions (Schwartz, 2008). The results showed that the number of TOTs decreased in the verbal WM condition, and the number of FOKs decreased in the visual WM condition, suggesting that temporal memory performance declined when participants made metacognitive judgments about general knowledge (Schwartz, 2008).

The present research examined how WMC limits affect metacognitive monitoring using a verbal working memory task (LST). Assuming overlap between object-level and meta-level processes based on PFC activity, individual differences in cognitive abilities should contribute to metacognitive judgment accuracy. Therefore, I confirmed the difference in monitoring accuracy between high and low WMC groups on target recall during the LST. WMC should influence workability and accuracy of metacognitive judgments due to monitoring. In addition, this research examined the interaction between memory load and interference from monitoring noise to investigate the factors that cause monitoring failures, especially underestimation of successful recall.

## Materials and methods

### Participants

Eighty-one Japanese undergraduate students participated in this research for partial credit in an experimental psychology course. Participants were all female and native speakers of Japanese with normal hearing. Based on LST scores, 25 participants were categorized as high WMC, and 25 were categorized as low WMC. Prior to WMC grouping, three participants were excluded for performance on the secondary comprehension task lower than 0.5. The average age of the 50 participants in the WMC groups was 20.58 years (*SD* = 0.79, range = 19–22).

To examine differences in cognitive and metacognitive performance due to WMC, participants were categorized into groups according to LST span and number of correctly recalled words. According to Conway et al. (2005), the group classification was based on quartile splits. The top quarter of the distribution (25 participants) had a span score of 4.0 or greater and 60 or more recalled words (span: *M* = 4.48, *SD* = 0.42; words: *M* = 63.08, *SD* = 2.60) and were categorized as high WMC; the bottom quarter of the distribution (25 participants) had span scores of 3.0 or less and 54 or less recalled words (span: *M* = 2.58, *SD* = 0.43; words: *M* = 47.56, *SD* = 5.16) and were categorized as low WMC. Mean accuracy on the secondary comprehension task (true/false decision) was 0.76 (*SD* = 0.07) and 0.73 (*SD* = 0.08) for the high and low WMC groups, respectively. A one sample *t*-test indicated that both scores were significantly greater than the chance level of 0.50 [high: *t*_(24)_ = 17.98, *p* < 0.001; low: *t*_(24)_ = 15.03, *p* < 0.001]. A student *t*-test indicated that the difference in true/false decision accuracy between high and low WMC groups was not significant [*t*_(48)_ = 1.53, *p* = 0.13].

All participants provided informed consent after they read an instruction sheet about the research purpose and participants' rights. The present research employed a behavioral experiment which does not require ethical approval. The procedure of this research did not include either invasiveness or intervention according to Ethical Guidelines for Medical and Health Research Involving Human Subjects, a Japanese guideline noticed by Ministry of Education, Culture, Sports, Science and Technology as well as Ministry of Health, Labour and Welfare.

### Materials

A Japanese version of the LST (Osaka et al., 2003) that included a primary word recall task and a secondary true/false decision task was used. Stimuli were 70 Japanese sentences with 35–46 moras (*M* = 41.77, *SD* = 2.64). There were five trials for each of four memory load conditions (two, three, four, or five sentences). Memory load conditions varied in the number of target words to be memorized, that is, the number of sentences processed within a trial. Table 1 shows an example from the two-sentence condition with the English translation. Target words were the first word of each sentence, which were all nouns. For the true/false decisions, 35 sentences were “true” (e.g., “Telephones are devices that encode voices as signals to communicate with a distant person and include mobile-phones”), and the other 35 sentences were “false” (e.g., “When you make scissors with the right hand and paper with the left hand while playing rock-paper-scissors, the number of folded fingers is two;” the correct number is three).

**Table 1:** **Sample sentences in the two-sentence condition of the Japanese Listening Span Test**.

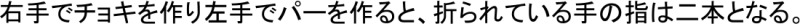	
When you make scissors with the right hand and paper with the left hand while playing rock-paper-scissors, the number of folded fingers is two.	
Target word: 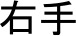 (right hand)	T/F decision: false
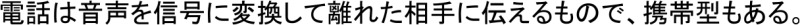	
Telephones are devices that encode voices as signals to communicate with a distant parson, and include mobile phones.	
Target word: 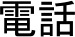 (telephones)	T/F decision: true

### Procedure

**Figure 1:**
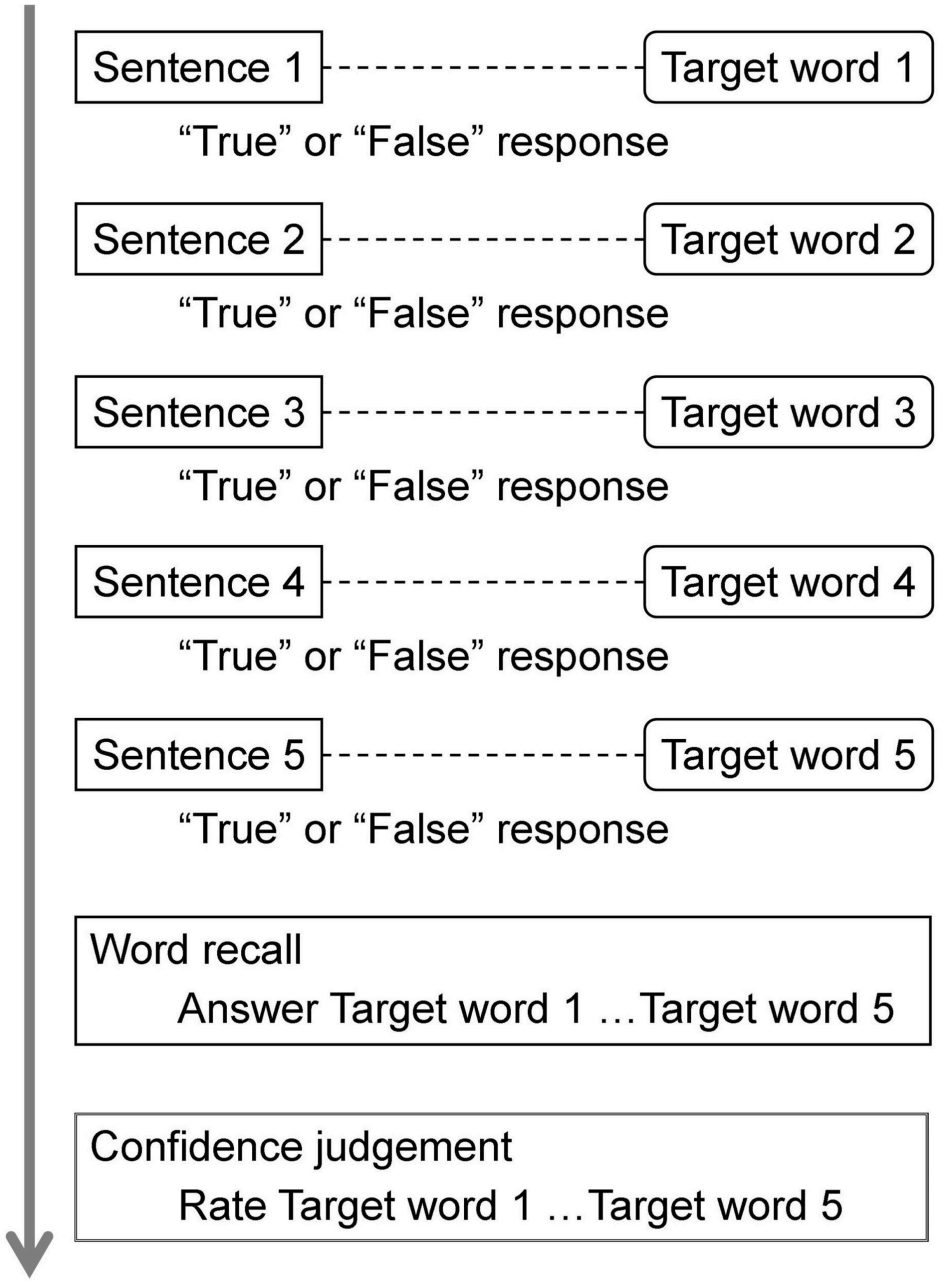
**Experimental procedure in the five-sentence condition**. On each trial, participants maintained the first word of each sentence, and judged whether each sentence was true or false based on general knowledge. Immediately after the experimenter finished reading a sentence, the true/false decision was written down. At the end of each trial, participants recalled every word they had memorized during that trial, and rated their retrospective confidence for recall performance for each word.

Participants were first instructed to listen carefully to a sequence of sentences read by a female experimenter. They were required to maintain the first word of each sentence, and, at the same time, judge whether each sentence was true or false based on general knowledge (see Figure 1). The number of sentences to be processed within a trial increased by one sentence every five trials. Immediately after the experimenter finished reading a sentence, the true/false decision was written down. At the end of each trial, participants were asked to recall every word they had memorized during that trial, and to rate their retrospective confidence for recall performance for each word from 0 to 100%. Although participants were able to recall target words in an arbitrary order within each trial, they were not allowed to recall the last target first. There were two practice trials in the two-sentence condition so that participants could learn the procedure.

The total number of correct words and span score were measured. Span was defined as the highest memory load condition level where the participant succeeded in three of five trials, with 0.5 points added for two successful trials in the following condition level. True/false decision accuracy was used as a measure of listening comprehension performance, and confidence for word recall performance was used as a measure of metacognitive monitoring.

## Results

### Listening span test

Before investigating effects of metacognitive level, effects on primary recall performance were examined using a mixed analyses of variances (ANOVA) with memory load condition (two, three, four, or five sentences) and true/false decision accuracy (correct vs. incorrect) as within-subjects factors, and WMC group (high vs. low) as a between-subjects factor. Table 2 shows mean word recall accuracy for each memory load condition. A two-way mixed ANOVA with WMC group and memory load condition as factors revealed significant main effects of WMC group [*F*_(1, 48)_ = 144.55, *p* < 0.001, ηρ^*2*^ = 0.75] and memory load condition [*F*_(3, 144)_ = 60.99, *p* < 0.001, ηρ^*2*^ = 0.56], and a significant interaction [*F*_(3, 144)_ = 16.85, *p* < 0.001, ηρ^*2*^ = 0.26]. Multiple comparisons (Bonferroni-corrected) showed significant differences in all the six pairwise tests using the corrected critical *p*-value, 0.008 [two vs. three: *t*_(49)_ = 4.15, *p* < 0.001; two vs. four: *t*_(49)_ = 6.82, *p* < 0.001; two vs. five: *t*_(49)_ = 8.26, *p* < 0.001; three vs. four: *t*_(49)_ = 5.28, *p* < 0.001; three vs. five: *t*_(49)_ = 7.31, *p* < 0.001; four vs. five: *t*_(49)_ = 5.00, *p* < 0.001].

**Table 2:** **Mean word recall accuracy in each memory load condition for high and low WMC participants**.

	**2-sentence**		**3-sentence**		**4-sentence**		**5-sentence**	
	***M***	***SD***	***M***	***SD***	***M***	***SD***	***M***	***SD***
High WMC	0.96	0.08	0.96	0.06	0.91	0.07	0.84	0.07
Low WMC	0.91	0.12	0.74	0.13	0.66	0.08	0.57	0.13

*Post-hoc* one-way ANOVAs of the interaction showed significant simple main effects of WMC in the three- [*F*_(1, 48)_ = 56.82, *p* < 0.001, ηρ^*2*^ = 0.54], four- [*F*_(1, 48)_ = 142.35, *p* < 0.001, ηρ^*2*^ = 0.75], and five-sentence [*F*_(1, 48)_ = 89.67, *p* < 0.001, ηρ^*2*^ = 0.65] conditions. The effect of WMC group on target recall performance was not significant for the two-sentence condition [*F*_(1, 48)_ = 2.88, *p* = 0.10, ηρ^*2*^ = 0.06]. *Post-hoc* one-way ANOVAs for simple main effects of memory load also observed significant effects for both high [*F*_(3, 144)_ = 10.06, *p* < 0.01, ηρ^*2*^ = 0.30] and low [*F*_(3, 144)_ = 67.78, *p* < 0.001, ηρ^*2*^ = 0.74] WMC groups. Multiple comparisons (Bonferroni test, the corrected critical *p*-value 0.008) indicated that recall performance decreased as memory load increased in the low WMC group [two vs. three: *t*_(24)_ = 5.99, *p* < 0.001; two vs. four: *t*_(24)_ = 9.80, *p* < 0.001; two vs. five: *t*_(24)_ = 8.46, *p* < 0.001; three vs. four: *t*_(24)_ = 3.42, *p* < 0.001; three vs. five: *t*_(24)_ = 4.89, *p* < 0.001; four vs. five: *t*_(24)_ = 3.33, *p* < 0.005]. In contrast, in the high WMC group, accuracy was significantly lower in the five-sentence condition than in any other condition [vs. two: *t*_(24)_ = 5.37, *p* < 0.001; vs. three: *t*_(24)_ = 6.47, *p* < 0.001; vs. four: *t*_(24)_ = 3.95, *p* < 0.001], and there was also a significant difference in accuracy between the three- and four-sentence conditions [*t*_(24)_ = 3.03, *p* < 0.006]. Participants with high WMC did not show a significant difference in performance between the two-sentence condition and the three- [*t*_(24)_ = −0.81, *p* = 0.94] or four-sentence [*t*_(24)_ = 2.27, *p* = 0.03] conditions.

Next, mean accuracy in the primary word recall task was compared between correct and incorrect true/false decisions in the secondary sentence comprehension task (See Table 3). A two-way mixed ANOVA indicated significant main effects of WMC group [*F*_(1, 48)_ = 92.62, *p* < 0.001, ηρ^*2*^ = 0.66] and true/false decision accuracy [*F*_(1, 48)_ = 13.15, *p* < 0.001, ηρ^*2*^ = 0.22]. There was no significant interaction between WMC group and secondary task accuracy, suggesting that incorrect sentence verification interfered with remembering targets regardless of WMC.

**Table 3:** **Mean word recall accuracy for correct and incorrect true/false decisions for high and low WMC participants**.

	**Correct decisions**		**Incorrect decisions**	
	***M***	***SD***	***M***	***SD***
High WMC	0.92	0.05	0.84	0.11
Low WMC	0.71	0.10	0.62	0.17

### Metacognitive monitoring

Retrospective confidence for each recalled word was rated from 0 to 100% as a measure of metacognitive monitoring of LST performance accuracy. Confidence percentage scores were converted to decimal scores to calculate metacognitive judgment outcome scores (see Table 4). The following four indexes were adopted based on Schraw (2009). Absolute Accuracy assesses the precision of a confidence judgment with respect to actual performance, and is calculated by the formula below, where *N* equals the total number of confidence judgments, *c*_*i*_ corresponds to a confidence rating, and *p*_*i*_ corresponds to a performance score (Schraw, 2009, p. 36, formula 1):

$$\text{Absolute Accuracy Index }=\;\frac{1}{N}\underset{i=1}{\sum^{N}}{\left(c_{i}-p_{i}\right)}^{2}$$

**Table 4:** **Mean confidence in overall word recall performance and monitoring index scores for high and low WMC participants**.

	**High WMC**		**Low WMC**	
	***M***	***SD***	***M***	***SD***
Confidence	89.03	12.51	81.79	14.55
Absolute accuracy	0.06	0.06	0.12	0.07
Discrimination	0.80	0.15	0.71	0.17
Bias	−0.07	0.13	−0.06	0.12
Scatter	0.02	0.03	0.02	0.02

Bias refers to the direction and magnitude of overconfidence or underconfidence (Schraw, 2009, p. 37, formula 3):

$$\text{Bias Index }=\;\frac{1}{N}\underset{i=1}{\sum^{N}}\left(c_{i}-p_{i}\right)$$

Scatter indicates the degree to which judgments for correct and incorrect responses differ in variability (Schraw, 2009, p. 37, formula 4):

$$\text{Scatter Index }=\;\frac{1}{N}\left[N_{c}\;var\left(c_{correct}\right)-N_{i}\;var\left(c_{incorrect}\right)\right]$$

Here, *N*_*c*_ and *N*_*i*_ indicate the number of correct and incorrect items, respectively, and *var* (*c*_*correct*_) and *var* (*c*_*incorrect*_) indicate variance in confidence judgments for correct and incorrect items, respectively. Finally, Discrimination reflects an individual's ability to distinguish between correct and incorrect outcomes (Schraw, 2009, p. 37, formula 5):

$$\text{Discrimination Index }=\;\frac{1}{N}\left[\underset{i=1}{\sum^{N_{c}}}{\left(c_{i\;correct}\right)}_{c}-\underset{i=1}{\sum^{N_{i}}}\left(c_{i\;incorrect}\right)\right]$$

Here, *c*_*i correct*_ and *c*_*i incorrect*_ correspond to confidence for correct and incorrect items, respectively. Relative accuracy (Schraw's formula 2) was not included here because participants only rated confidence for individual trials, not the whole task.

For each measurement (see Table 4), the difference between high and low WMC groups was examined with student *t*-tests. The mean confidence score for recalled words was numerically higher for the high vs. low WMC group, but the difference was not significant [*t*_(48)_ = 1.86, *p* = 0.65]. Absolute Accuracy was significantly smaller in the high vs. low WMC group [*t*_(48)_ = −3.04, *p* < 0.01]. The high WMC group judged their performance more precisely than the low WMC group (Absolute Accuracy scores of zero correspond to perfect accuracy).

Mean Discrimination scores were positive for both WMC groups. A one sample *t*-test indicated Discrimination scores in both groups were reliably different from zero [high: *t*_(24)_ = 26.09, *p* < 0.001; low: *t*_(24)_ = 20.95, *p* < 0.001], suggesting participants were more confident in correct vs. incorrect responses. In addition, the discrepancy magnitude of Discrimination was significantly larger in the high vs. low WMC group [*t*_(48)_ = 2.02, *p* < 0.05].

Bias scores were negative for both groups. One sample *t*-tests showed that Bias scores for both groups were significantly different from zero [high: *t*_(24)_ = −2.83, *p* < 0.01; low: *t*_(24)_ = −2.63, *p* < 0.05], indicating that participants were underconfident in their performance. The group difference in Bias was not significant [*t*_(48)_ = −0.22, *p* = 0.82]. Scatter indices did not differ between groups [*t*_(48)_ = −0.01, *p* = 0.996].

### Underestimation of metacognitive judgments

Because participants underestimated successful memory performance, two-way mixed analyses of variances (ANOVA) tested confidence scores for correct word recall only, with memory load condition (two, three, four, or five sentences), secondary true/false decision accuracy (correct vs. incorrect), and ongoing trial accuracy (correct vs. incorrect) as within-subjects factors, and WMC group (high vs. low) as a between-subjects factor. To examine possible factors that interfere with metacognitive monitoring, *post-hoc* analyses were conducted for simple main effects even when interactions were not statistically significance at *p* < 0.05.

#### Memory load

Mean confidence scores for high and low WMC groups were examined in each memory load condition (see Table 5). A two-way mixed ANOVA with WMC group and memory load condition as factors showed a significant main effect of memory load [*F*_(3, 144)_ = 6.61, *p* < 0.001, ηρ^*2*^ = 0.12]; neither the main effect of WMC group [*F*_(1, 48)_ = 9.08, *p* = 0.09, ηρ^*2*^ = 0.06] nor the interaction between WMC group and load [*F*_(3, 144)_ = 0.56, *p* = 0.65, ηρ^*2*^ = 0.01] were significant. Multiple comparisons (Bonferroni test, the corrected critical *p*-value 0.008) indicated that confidence was significantly lower in the five-sentence condition compared to the three- [*t*_(49)_ = 4.05, *p* < 0.001] and two-sentence [*t*_(49)_ = 3.33, *p* < 0.005] conditions. There was no significant difference between any other pair of load conditions [two vs. three: *t*_(49)_ = 0.23, *p* = 0.82; two vs. four: *t*_(49)_ = 1.29, *p* = 0.20; three vs. four: *t*_(49)_ = 1.26, *p* = 0.21; four vs. five: *t*_(49)_ = 2.48, *p* = 0.02].

**Table 5:** **Mean confidence in correct word recall in each memory load condition for high and low WMC participants**.

	**2-sentence**		**3-sentence**		**4-sentence**		**5-sentence**	
	***M***	***SD***	***M***	***SD***	***M***	***SD***	***M***	***SD***
High WMC	90.88	11.68	91.04	11.45	90.69	12.87	85.70	17.98
Low WMC	85.37	15.58	84.69	18.20	81.75	14.81	79.37	15.63

Further one-way ANOVAs of WMC effects revealed a significant simple main effect in the four-sentence condition [*F*_(1, 48)_ = 5.19, *p* < 0.05, ηρ^*2*^ = 0.10], and no significant effect in the two- [*F*_(1, 48)_ = 2.00, *p* = 0.16, ηρ^*2*^ = 0.04], three- [*F*_(1, 48)_ = 2.18, *p* = 0.15, ηρ^*2*^ = 0.04], or five-sentence [*F*_(1, 48)_ = 1.77, *p* = 0.19, ηρ^*2*^ = 0.04] conditions. The *post-hoc* one-way ANOVAs indicated simple main effects of memory load condition were significant for both high [*F*_(3, 144)_ = 3.34, *p* < 0.05, ηρ^*2*^ = 0.12] and low [*F*_(3, 144)_ = 3.83, *p* < 0.05, ηρ^*2*^ = 0.14] WMC groups. Multiple comparisons (Bonferroni test, the corrected critical *p*-value 0.008) indicated a significant difference between the five- vs. three-conditions in the high WMC group [*t*_(24)_ = 3.50 *p* < 0.005)]. There was no significant difference between any other pair of load conditions in the high WMC group [two vs. three: *t*_(24)_ = −0.13, *p* = 0.98; two vs. four: *t*_(24)_ = 0.08, *p* = 0.94; two vs. five: *t*_(24)_ = 2.29, *p* = 0.03; three vs. four: *t*_(24)_ = 0.20, *p* = 0.84; four vs. five: *t*_(24)_ = 2.04, *p* = 0.05] or the low WMC group [two vs. three: *t*_(24)_ = 0.35, *p* = 0.73; two vs. four: *t*_(24)_ = 1.90, *p* = 0.07; two vs. five: *t*_(24)_ = 2.38, *p* = 0.03; three vs. four: *t*_(24)_ = 1.51, *p* = 0.14; three vs. five: *t*_(24)_ = 2.44, *p* = 0.02; four vs. five: *t*_(24)_ = 1.40, *p* = 0.18].

#### Noise from the secondary task

To examine effects of additional information from the secondary true/false task, confidence for correct word recall was compared between correct vs. incorrect sentence comprehension (see Table 6). A two-way mixed ANOVA indicated a significant main effect of true/false accuracy [*F*_(1, 48)_ = 14.87, *p* < 0.001, ηρ^*2*^ = 0.24]. The main effect of WMC group [*F*_(1, 48)_ = 2.85, *p* = 0.10, ηρ^*2*^ = 0.06] and the interaction between WMC and true/false accuracy [*F*_(1, 48)_ = 3.15, *p* = 0.08, ηρ^*2*^ = 0.06] were not significant.

**Table 6:** **Mean confidence in correct word recall for correct and incorrect true/false decisions for high and low WMC participants**.

	**Correct decisions**		**Incorrect decisions**	
	***M***	***SD***	***M***	***SD***
High WMC	90.72	11.86	88.67	14.96
Low WMC	85.69	14.76	80.16	16.47

Simple effects for the interaction of interest were examined using *post-hoc* one-way ANOVAs. Low WMC participants showed a significant decrease in confidence for incorrect true/false decisions despite their accurate word recall [*F*_(1, 48)_ = 15.86, *p* < 0.001, ηρ^*2*^ = 0.40], whereas high WMC participants did not show a significant reduction in confidence based on secondary task accuracy [*F*_(1, 48)_ = 2.16, *p* = 0.15, ηρ^*2*^ = 0.09]. The *post-hoc* one-way ANOVAs also showed a marginal difference between WMC groups for incorrect true/false decisions [*F*_(1, 48)_ = 3.66, *p* = 0.06, ηρ^*2*^ = 0.07]. This difference was not significant for correct true/false decisions [*F*_(1, 48)_ = 1.76, *p* = 0.19, ηρ^*2*^ = 0.04].

#### Noise from the ongoing trial

Table 7 shows confidence for correct word recall on correct and incorrect trials. A two-way ANOVA showed a significant main effect of trial accuracy [*F*_(1, 48)_ = 13.65, *p* < 0.001, ηρ^*2*^ = 0.22]. The main effect of the WMC group [*F*_(1, 48)_ = 1.36, *p* = 0.25, ηρ^*2*^ = 0.02] and the interaction [*F*_(1, 48)_ = 0.15, *p* = 0.70, ηρ^2^ = 0.003] were not significant. Participants rated their confidence for correct answers significantly lower due to other failures within the ongoing trial, irrespective of WMC.

**Table 7:** **Mean confidence in correct word recall for correct and incorrect trials for high and low WMC participants**.

	**Correct trials**		**Incorrect trials**	
	***M***	***SD***	***M***	***SD***
High WMC	91.59	11.23	87.63	16.84
Low WMC	87.41	13.76	82.54	16.33

Further analysis of interest using *Post-hoc* one-way ANOVAs indicated significant simple main effects of trial accuracy for both WMC groups [high: *F*_(1, 48)_ = 5.49, *p* < 0.05, ηρ^*2*^ = 0.19; low: *F*_(1, 48)_ = 8.31, *p* < 0.01, ηρ^*2*^ = 0.24]. While, simple main effects of WMC by *post-hoc* one-way ANOVAs were not significant [correct: *F*_(1, 48)_ = 1.39, *p* = 0.24, ηρ^*2*^ = 0.03; incorrect: *F*_(1, 48)_ = 1.18, *p* = 0.28, ηρ^*2*^ = 0.03].

## Discussion

The current research examined metacognitive monitoring when WMC was limited. Participants had to maintain the first word of each sentence in a sequence of spoken sentences (primary task), and, at the same time, verify whether each sentence was true or false according to general knowledge (secondary task). Immediately after each sentence was read, participants made true/false decisions, and then recalled all target words at the end of the trial. Metacognitive monitoring was measured by ratings of retrospective confidence for each word at recall.

First, this research confirmed that WMC affects absolute accuracy of metacognitive judgments in a multi-task situation: participants with high WMC were better at discriminating between correct and incorrect recall, and their confidence ratings were more consistent with their actual performance than low WMC participants. Further examination of the underconfidence bias showed that increased memory load and interference from task-irrelevant information (inter- or inner-task noise) interfered with monitoring accuracy. These interference effects suggest differences in the ability to focus on monitoring outcomes between high and low WMC participants.

To explain the dissociation between confidence and performance, recent metacognitive studies have adopted inference-based rather than direct-access approaches, suggesting that confidence judgments should depend on peripheral mnemonic cues such as learning and response latencies (Schwartz, 1994; Dunlosky and Metcalfe, 2009; Koriat, 2012). According to Koriat (2012), for general knowledge questions, “confidence and its accuracy appear to depend in part on the on-line feedback from the process of answering a question or solving a problem” (p. 7). However, it seems inappropriate to understate the value of direct access to metacognition, as the LST requires that participants retrieve memory traces of target words from WM representations. Therefore, according to recent frameworks for WM, accuracy of metacognitive judgments during a verbal WM task may depend on the ability to find effective trace cues within primary memory and to ignore goal-irrelevant mnemonic cues from secondary memory.

### Individual differences in monitoring accuracy

Differences in people's cognitive abilities derived from WMC have been explained by attentional control processes (allocation: e.g., Just and Carpenter, 1992; focusing: e.g., Baddeley, 2007; Osaka et al., 2007; maintenance and inhibition of proper information: e.g., Engle and Kane, 2004; Unsworth and Engle, 2007; Miyake and Friedman, 2012; scope and control: e.g., Chow and Conway, 2015) based on the underlying assumption that monitoring is critical. Such attentional theories are supported by neurocognitive evidence from fMRI studies, which suggest that the basis of individual (group) differences in WMC involve neural networks that include the DLPFC for updating information, the ACC for monitoring conflicts (MacDonald et al., 2000; Osaka et al., 2003, 2004; Shimamura, 2008), and the SPL for focusing (Osaka et al., 2007). In observing the cooperative activation of these regions in high WMC participants during the RST, N. Osaka explained, “…this network was active in monitoring the task performance, which probably helped their task performance effectively” (Osaka et al., 2004, p. 8). Supporting this assumption, the results of the present research confirm significant differences in monitoring ability associated with WMC.

Considered within the recent WM frameworks, proper monitoring during a cognitive activity can facilitate detecting differences between ongoing task performance and task goal maintained in primary WM storage (Unsworth and Engle, 2007), and then regulate subsequent processes via feedback from monitoring outcomes. Concurrently, monitoring might trigger the gating function of updating-specific EF (Miyake and Friedman, 2012) to inhibit unnecessary information (Engle and Kane, 2004; Kane et al., 2007) and focus on goal-related information (Osak et al., 2002) based on perceived conflicts between representations within primary WM or interference from task-irrelevant information in secondary WM (Unsworth and Engle, 2007).

Although, low WMC impaired absolute monitoring accuracy, low WMC did not block monitoring completely. The positive discrimination scores in both WMC groups show that participants were able to monitor their memory processes even in the multi-task situation where cognitive resources were in short supply, and to distinguish their recall success and failure retrospectively. Participants were instructed to rate their confidence in each recalled word before the experiment so that they could intentionally monitor their performance. In addition, confidence judgments were made immediately after recalling all the targets within the recall phase of that trial. Instant re-access to WM representations for metacognitive judgments after recollection makes it possible for even low WMC participants to detect whether target information remained active within WM, and then to translate particular signal strength associated with a memorized target word into a confidence rating. This is consistent with the direct-access theory of metacognitive judgments that assumes that the existence and strength of memory traces are crucial cues to confidence (for reviews see Schwartz, 1994; Dunlosky and Metcalfe, 2009; Koriat, 2012).

### Factors that contribute to underconfidence

The results of the present research show that memory load may be one cause of the underconfidence bias. In both WMC groups, confidence judgments mirrored the pattern of actual performance across memory load conditions, although the interaction between WMC group and memory load condition was more prominent for cognitive performance. For participants with high WMC, accuracy and confidence judgments decreased for five-sentence trials. For participants with low WMC, cognitive and metacognitive performance gradually decreased as memory load increased. In addition, high WMC participants tended to make more accurate metacognitive judgments than low WMC participants in the four-sentence condition, which also showed the largest group difference in memory performance.

Schwartz (2008) also found decreases in monitoring accuracy by WM load: TOT judgments interfered with a verbal WM task, and FOK judgments interfered with a visual WM task. This suggests that WM and metacognitive monitoring share processes and resources with respect to retrieval and attentional allocation (for TOT, see Schwartz and Metcalfe, 2011). According to the direct-access approach to metacognition, these results suggest that WM load weakens target traces because of deficiency in the shared resources among meta-level and object-level processes. The weakened target trace signal might be responsible for underconfidence in correct word recall in the present research, and the absence of TOT in Schwartz (2008).

It is also possible to interpret the effect of memory load in the current research according to the inference-based view, that is, as a peripheral cue about task difficulty. Participants perceived increasing task difficulty in accordance with their decrease in memory performance, reflecting precise monitoring of overall cognitive processes. Thus, they may underestimate confidence for correct answers when attention is focused on experience-based cues of task difficulty rather than direct cues from target traces.

Other factors that may cause an underconfidence bias are monitoring noise derived from the secondary sentence verification task, and from within the primary memory task trial. These both depend on experience-based cues of difficulty or negative feelings from partial LST performance failure, but they differ in the noise source. First, participants underestimated their confidence for correctly recalled words when they failed to make correct decisions in the secondary task. In that case, participants may have emphasized task difficulty and negative feelings in metacognitive judgments, monitoring their effortful or unsuccessful experiences in the secondary verification task. Furthermore, this underconfidence bias due to external noise was stronger for low vs. high WMC participants. High WMC may involve simultaneously inhibiting irrelevant monitoring information from secondary memory and focusing on important monitoring cues from target traces within primary memory. Proper monitoring function is reflected in cognitive performance: high WMC participants recalled more target words than low WMC participants when secondary task performance was incorrect, even though they made as many mistakes in the secondary task as low WMC participants.

Second, monitoring noise from the primary trial elicited an underconfidence bias when participants partly failed to recall target words within the current memory task trial. When target words were retrieved, participants accessed the representation in primary WM to detect all target traces for the current trial. Then, they made responses according to memory trace signal strength. Although, participants could distinguish between correct and incorrect answers by monitoring memory traces, an absent or weak trace signal may have impaired confidence for correctly reported words. Unlike noise from the secondary task, interference from failure within the primary task did not contribute to individual differences. It appears difficult to inhibit noise information and separate target representations from integrated sub-processes within a trial independently of WMC.

It is beneficial to identify which cues to be inhibited and which to be focused because the use of proper monitoring cues can facilitate cognitive performance. Previous research that manipulated the consistency between the target word for maintenance and the focus word for comprehension in the RST showed that word recollection was facilitated in low WMC readers when they were able to focus attention on a single target-and-focus word in each sentence (Osak et al., 2002; Osaka et al., 2007). This directing effect on attentional control should translate to metacognitive monitoring by manipulating important mnemonic cues. For example, the present study suggests that emotional cues may be promising: negative emotional experience evoked by partial task failure impaired monitoring accuracy. Moreover, Eysenck and Calvo (1992) proposed that anxiety impedes the efficiency of EFs because task-irrelevant thoughts occupy limited processing resources (for a review, see Derakshan and Eysenck, 2009). In addition, the emotional value of stimuli facilitates WM in some cases, but impairs it in others (e.g., Kensinger and Corkin, 2003). More data are needed to clarify what kind of information is effective as a monitoring cue from the viewpoint of individual differences in monitoring accuracy.

In conclusion, the results of the present research confirmed individual (group) differences in WMC in metacognitive monitoring abilities. It also suggested that high WMC helps inhibit irrelevant monitoring noise and focus on detecting target traces or useful retrieval cues, which improves actual cognitive performance. Future studies are needed to reveal more details of monitoring processes and individual differences in monitoring abilities.

## Author contributions

The author confirms being the sole contributor of this work and approved it for publication.

### Conflict of interest statement

The author declares that the research was conducted in the absence of any commercial or financial relationships that could be construed as a potential conflict of interest.
